# Characterizing the complexity of enzymes on the basis of their mechanisms and structures with a bio-computational analysis

**DOI:** 10.1111/j.1742-4658.2011.08190.x

**Published:** 2011-10

**Authors:** Gemma L Holliday, Julia D Fischer, John B O Mitchell, Janet M Thornton

**Affiliations:** 1 EMBL-EBI, Wellcome Trust Genome CampusCambridge, UK; 2 Biomedical Sciences Research Complex and EaStCHEM School of Chemistry, University of St AndrewsUK

**Keywords:** active sites, catalysis, enzyme, evolution, MACiE, mechanism, specificity, structure

## Abstract

Enzymes are basically composed of 20 naturally occurring amino acids, yet they catalyse a dizzying array of chemical reactions, with regiospecificity and stereospecificity and under physiological conditions. In this review, we attempt to gain some understanding of these complex proteins, from the chemical versatility of the catalytic toolkit, including the use of cofactors (both metal ions and organic molecules), to the complex mapping of reactions to proteins (which is rarely one-to-one), and finally the structural complexity of enzymes and their active sites, often involving multidomain or multisubunit assemblies. This work highlights how the enzymes that we see today reflect millions of years of evolution, involving *de novo* design followed by exquisite regulation and modulation to create optimal fitness for life.

## Introduction

Enzymes are protein polymers that catalyse biochemical reactions and, without them, life as we know it could not exist. Although enzymes are the most prominent of biological catalysts, examples of RNA catalysts (termed ribozymes) have been found. However, these are outwith the scope of this review. These proteins are formed from a pool of the 20 standard amino acids plus the rarer selenocysteine and l-pyrrolysine, which are encoded in the genetic code of life. From these basic building blocks, enzymes have evolved to perform the vast repertoire of chemical reactions, many of which are highly complex, found in nature. They do this under physiological conditions (around pH 7, 1 atm and in aqueous solution) with phenomenal yields and exquisite stereoselectivity and regioselectivity, a continuing goal for many synthetic organic chemical processes, which often need very harsh conditions to perform the same chemistry. Enzymes are also responsible for the uptake, synthesis and breakdown of chemicals, such as drugs or environmental contaminants (e.g. pesticides), in our bodies. In humans, thousands of enzymes control the rates of essential cellular reactions, and enzymes represent ∼ 63% of all drug targets (of the 3832 targets annotated in ChEMBL, 2443 are associated with enzymes) [1]. Enzymes are also vastly complex molecules with respect to their quaternary structure. Although some are simple, being only a single domain on a single chain and tens of kilodaltons in molecular mass (e.g. dihydrofolate reductase, which is ∼ 18 kDa in molecular mass), others are vast (e.g. plant ribulose-1,5-bisphosphate carboxylase oxygenase, which is composed of eight copies of a large protein chain and eight copies of a smaller chain, giving a total molecular mass of ∼ 540 kDa).

Although the mechanisms of several hundred enzymes have been fully characterized experimentally and are well understood, they represent only a small fraction of the total number of enzymes found in nature. All characterized enzymes have an Enzyme Commission (EC) number [2,3], a code that has long been used to classify enzymes with respect to the overall transformation of substrate into product, and that uses a four-level description. The first three levels (class, subclass and sub-subclass) broadly define the overall chemistry occurring, and the serial number (the fourth level) generally defines the substrate specificity. To date (May 2011), there are 4444 enzyme reactions classified by the EC, and this number is steadily growing. However, the mechanisms of many of these enzymes are hypothetical or poorly understood (at best), and we still do not fully understand how enzymes manage to catalyse such a huge number of different chemical reactions with such a limited repertoire of chemical building blocks, the 20 amino acids (of which only 10 are, commonly, directly involved in catalysis), with the help of some post-translational modifications, 27 small-molecule organic cofactors [4,5] and the 13 metal ions [6,7].

MACiE [8,9,10] (http://www.ebi.ac.uk/thornton-srv/databases/MACiE), a database of distinct enzyme mechanisms, was thus created in order to enable a better understanding of how enzymes perform this array of chemical reactions with such exquisite accuracy, and provides the basic data for the work presented herein.

MACiE has been populated (to date) with enzymes that are unique at the mechanism level (number and order of steps, catalytic site, including amino acids and cofactors, and the chemical changes involved), rather than nonhomologous at the evolutionary level, and the current version (2.5) contains 280 unique mechanisms covering 268 distinct EC numbers. MACiE is a valuable tool with which to advance our understanding of the chemistry of enzyme reaction mechanisms [6,11–16], as well as potential applications in the area of protein design [17]. Although there is a plethora of knowledge, including structures, gene sequences, mechanisms, metabolic pathways and kinetic data, it is spread between many different databases and throughout the literature (a Medline search for ‘enzyme’ and ‘mechanism’ produces over 200 000 hits and over 18 000 reviews). Although there are many resources (e.g. UniProtKB [18], wwPDB [19], IntEnz [2], ExplorEnz [3], pFam [20], BRENDA [21], PLD [22], KEGG [23], BioPath [24], Promise [25] and MDB [26]) that describe the overall chemistry, MACiE is unique in combining detailed stepwise mechanistic information while trying to cover as much of the chemical space and the protein structure universe as possible. Worldwide, there is increasing interest in compiling such mechanistic information for enzymes, and MACiE usefully complements both the mechanistic detail of the Structure–Function Linkage Database [27], which provides great detail for a small number of enzyme superfamilies, and the wider coverage with less chemical detail provided by EzCatDB [28], which also contains a limited number of 3D animations and the Catalytic Site Atlas (CSA) [29].

## Chemical diversity of the catalytic toolkit – amino acids and cofactors

The catalytic site is the enzyme’s workshop, and it is here that the catalytic reaction occurs. This cleft, which is often buried (sometimes deeply) [30], houses a relatively small number of amino acids that are involved in binding the substrate (and/or cofactor), and an even smaller subset of these that are vital to the enzyme’s catalytic function.

In order to both study and understand the role and function of the catalytic amino acids in enzymes, it was first necessary to define what is meant by a ‘catalytic residue’. In MACiE, we have taken the definition first proposed by Bartlett *et al.* [30] and split that definition into two general categories, such that a catalytic residue is any residue involved in the reaction that: (a) has direct involvement in the reaction mechanism, the so-called reactant residues whose chemical structure is modified during the course of the reaction (for example, the residue is involved in covalent catalysis, electron shuttling or proton shuttling); and (b) has indirect, but essential, involvement in the reaction mechanism, the so-called spectator residues, whose chemical structure does not change during the course of the reaction – these are the residues that polarize or alter the p*K*_a_ of a residue, a water molecule or part of the substrate directly involved in the reaction, affect the stereospecificity or regiospecificity of the reaction, or stabilize the reactive intermediates (either by stabilizing the transition states or the intermediates themselves, or destabilizing the ground states of the substrates).

**Fig. 1:**
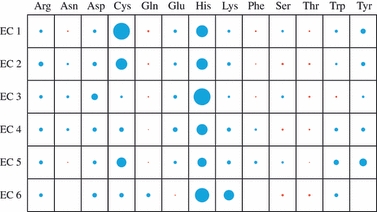
Balloon plot showing the propensity of a residue to be catalytic in each of the six classes of enzyme (EC 1, oxidoreductases; EC 2, transferases; EC 3, hydrolases; EC 4, lyases; EC 5, isomerases; EC 6, ligases). The diameter of the circle represents the value of the propensity; thus, the larger the circle, the higher the propensity of the residue to be catalytic. The circle is shown in blue if the propensity is greater than (or equal to) 1, and red if the propensity is < 1 (see Table S1 for exact values).

All 20 amino acids (Arg, Asp, Cys, Glu, His, Lys, Ser, Thr, Trp, Tyr, Asn, Gln, Ile, Leu, Pro, Gly, Ala, Phe, Met and Val) are seen in MACiE as part of the active site machinery. MACiE does not currently contain any entries that utilize either selenocysteine or pyrrolysine as catalytic residues, so we cannot determine whether this is attributable to a lack of activity or of annotation for these two residues. The nonpolar residues (Ile, Leu, Pro, Gly, Ala, Phe, Met, Val and Trp) very rarely act through their side chains; instead, they act mainly through their main chain portions (usually as either the N-H group or the C=O group). However, only 10 of the catalytic residues annotated in MACiE (Arg, Asp, Cys, Glu, His, Lys, Ser, Thr, Trp and Tyr) are absolutely essential [12,13], in that they perform almost all of the functions associated with catalysis in all classes of enzymes. Nonetheless, their prevalence as catalytic entities in the six different classes of enzyme clearly differs. Catalytic propensity is a measure of how often a residue is catalytic as compared with its background levels in a protein; thus, it is calculated by dividing the percentage of that residue type that is catalytic by the total percentage of that residue in the whole protein dataset. If the propensity is < 1, then the propensity for that residue to be catalytic is less than expected, and if it is > 1, then the residue is more catalytic than might be expected by chance. Figure 1 shows the catalytic propensities of the 10 residues that are most commonly catalytic [13], along with Asn, Gln and Phe.

What is immediately clear is that whereas some residues (such as His and Cys) are strongly catalytic in all classes, the catalytic propensities of the different residues varies between the six functional classifications of the EC. This difference is not attributable to differences between the background amino acid compositions of these enzymes (a detailed analysis is available from the database analysis and statistics section on the MACiE website). The differences in catalytic propensity are further seen in the functions that the residues are carrying out in the six different enzyme classes (as defined by the EC). It is possible to split the functions that the catalytic residues are performing into seven categories: (a) activation – residues that are responsible for activating other species; (b) steric role – residues that affect the outcome of the reaction through steric considerations; (c) stabilization – residues that (de)stabilize other species; (d) proton shuttling – residues that donate, accept or relay protons; (e) hydrogen radical shuttling – residues that donate, accept or relay hydrogen atoms; (f) electron shuttling – residues that donate, accept or relay electrons, either singly or in pairs; and (g) covalent catalysis – residues that become covalently attached to a reaction intermediate.

We have previously shown [13] that, with the exception of hydrogen radical shuttling and covalent catalysis (to a lesser degree), all of the residues examined are capable of performing all of the seven categories of residue function to some extent. However, the functional profiles of the residues analysed are different in each of the six enzyme (EC) classes, suggesting that the propensity of the residues to be catalytic in the different EC classes could well be related to the different roles that the residues can play. However, it is still not clear why residues that are capable of performing any one of the seven categories of function annotated have a predilection for performing certain functions in one class and other functions in another. We are currently looking into this phenomenon, including the effect of the local environment and physicochemical properties of these residues, in more detail.

## Extending the catalytic toolkit through cofactors

Amino acids are not the only catalytic entities in the active site. Cofactors, both metal ions and small organic molecules, offer an extension of the catalytic power of enzymes. Recently, we have extended the MACiE database to include Metal-MACiE [7,31], in order to fully categorize and annotate the metal ions in MACiE, and their roles and functions. We have also created CoFactor [4,5], in order to catalogue the organic, small-molecule cofactors in enzyme reactions.

As has been recently shown [16], there are certain functional roles that all three types of catalytic entity perform, although to greatly differing degrees. Generally speaking, metal ions are more active than either amino acids or organic cofactors in the area of electrostatic stabilization, and are more active in the role of electron shuttling (both pairs and singly) than amino acids, although not organic cofactors. The most striking difference in the functional roles played by these three entities is in the role of hydride shuttling, which is only performed by organic cofactors in this dataset. This suggests that amino acids and biologically active metal ions are not able (or at the very least are exceedingly unlikely) to perform this function. This hypothesis is further borne out by the overrepresentation of cofactor dependence in the oxidoreductase class, in which over 80% of enzymes require an organic cofactor, many of which are responsible for hydride shuttling [FAD, FMN and NAD(P)^+^].

## Mapping enzyme reactions to protein families

An enzyme is composed not only of the catalytic components (residues and cofactors) but also of many amino acids that make up the protein’s functional biological unit. It is worth noting that, in protein crystallography, the structure is determined in a crystalline environment, which may not reflect the functional biological unit *in vivo*; thus, the biological unit must be derived from the data in the Protein Data Bank (PDB) and associated biochemical information.

**Fig. 2:**
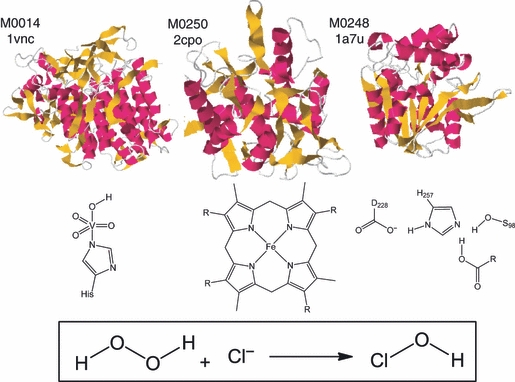
The complexity of chloroperoxidases (EC 1.11.1.10). The protein on the far left is the vanadate-dependent chloroperoxidase (MACiE entry M0014), the central protein is the haem-dependent enzyme (M0250), and the far right protein is the so-called cofactor-free enzyme (M0248). The top panel shows the protein structures and the related database codes, the middle section shows the main catalytic entities involved, and the bottom section shows the overall chemical transformation that all three enzymes perform, the formation of hypohalous acid from hydrogen peroxide and a halide ion.

In understanding the relationship between structure and function, we must map an enzyme reaction (as defined by the EC) to specific proteins, as defined by their sequences and structures. This mapping is complex and is rarely 1 : 1. Some enzymes perform multiple reactions; some reactions are performed by multiple unrelated enzymes. In addition, different enzymes with an identical EC number (and therefore overall reaction) occasionally have significantly different mechanisms. A good example of this phenomenon is provided by the haloperoxidases (EC 1.11.1.10). There are three different types of this enzyme that have been identified so far, all of which catalyse the same basic reaction (shown in the bottom panel of Fig. 2), and which can be classified on the basis of their cofactor dependence: (a) the vanadate-dependent enzyme, in which the hydrogen peroxide becomes bound to the vanadate cofactor and is ultimately eliminated as the hypohalous acid – the mechanism for this enzyme can be seen in MACiE entry M0014, and the protein structure can be represented by PDB code 1vnc [32–35]. (b) the haem-dependent enzyme, in which the hydrogen peroxide becomes bound to the haem cofactor, and the reaction proceeds via a radical mechanism – this enzyme can be seen in MACiE entry M0250, and the protein structure can be represented by PDB code 2cpo [36–38]; and (c) the so-called cofactor-free enzyme, which utilizes a Ser-His-Asp catalytic triad and a small organic acid to produce the reactive intermediate – this enzyme can be seen in MACiE entry M0248, and the protein structure can be represented by PDB code 1a7u [39].

As can be seen from Fig. 2, the three proteins also differ significantly in their protein structure, as well as in their chemical mechanisms, and they are evolutionarily unrelated.

In order to gain a better idea of the scope of this complexity and determine the total number of reactions *in vivo*, and thus the potential size of the MACiE database, we estimated the number of protein families per EC node on the basis of sequence data (for an upper limit) and structures (for a lower limit). Although this approach does not take into account that different EC nodes, especially within a sub-subclass, may have very similar mechanisms and relatives with respect to their evolution, the fact that they have different substrate specificities or different reaction outcomes (products) is considered to be important in this analysis. It is further worth noting that, as not all EC nodes have associated biological data (sequences with fully and unambiguously assigned EC nodes in Swiss-Prot or crystal structures in the PDB), the following can only be considered to be rough estimate.

## Enzyme-to-reaction mapping using sequences

It has previously been suggested that, whereas the theoretical limit on the size of protein space is astronomically large (thus, only an infinitesimally small portion of it has been explored during the course of life on earth), it is entirely feasible that most (if not all) of functionally relevant protein sequence space has already been explored [40]. Furthermore, enzymes that are homologous at the sequence level are likely to have the same (or at least a very similar) mechanism. Given this, we can make a guess at the total number of ‘known’ enzyme reaction mechanisms, on the basis of the sequence space currently annotated with a complete EC number. This, of course, does not include any reactions that have yet to be discovered; that is, we assume that unrelated enzymes will use different reaction mechanisms. We calculated the total number of enzyme protein sequence clusters that have been mapped to an EC number, and then, by dividing this by the total number of reactions, we calculated the number of protein sequence clusters associated with a given EC reaction (as defined by EC numbers).

We identified 2657 EC nodes (a fully defined EC number) that also had a sequence assigned (approximately half of all the currently defined EC nodes). For each of these EC nodes, we clustered the sequences using blastclust (part of the NCBI BLAST package), and identified a total of 13 150 sequence clusters, suggesting that there are, on average, 4.95 distinct evolutionary families per EC node. However, blastclust produces many singleton ‘clusters’, so the use of hidden Markov models allows many of these singleton clusters to be placed with other clusters. This gives a total of 9312 clusters, and an average of 3.50 distinct evolutionary families per EC node. That is, there are, on average, a little over three different ways in which the same overall chemistry can be performed. Almost 50% of EC nodes are only represented by a single evolutionary family (i.e. a single cluster), but this may be attributable to a lack of assigned sequences in the databases. The other half of the EC nodes all contain more than one family, with the nonspecific Ser/Thr protein kinase (EC 2.7.11.1) being the most promiscuous EC node, with 450 associated sequence families.

In order to compare these results more accurately with the structure analysis which follows, we can calculate the number of families per EC node for which both a sequence and a structure have been identified. Given that only approximately one-quarter of all EC numbers actually have a solved crystal structure deposited in the PDB, the number of families drastically decreases to an average of 2.02 evolutionary families per EC node.

## Enzyme-to-reaction mapping using structures

An enzyme is not just a sequence or an active site; it is a complete protein that can be composed of many different chains and structural domains. We can define a lower limit to the number of times that a reaction has evolved by utilizing the principle that, during protein evolution, two proteins may have significantly diverged in terms of sequence similarity, but may still have a common ancestor (and thus function), which can be identified through structural similarity, as this is maintained for much longer than sequence similarity [41].

Protein structure can be categorized in many different ways, but the two most common are CATH (Class, Architecture, Topology and Homologous superfamily) [42] and SCOP [43], and in this analysis we utilize the CATH code. CATH endeavours to identify cases in which the sequence similarity between two proteins is extremely low, but the structure-based analysis shows that they still retain sufficient similarity to indicate a common ancestor. The C, A and T levels of the CATH code basically define the structure, with the C level describing the secondary structure composition of each domain, the A level describing the shape revealed by the orientations of the secondary structure units, and the T level describing the sequential connectivity. When the structures belonging to the same T level have high similarities combined with similar functions, the proteins are assumed to have a common ancestor and are thus placed in the same H level. Thus, if an enzyme has an identical CATH code to a second enzyme, there is a good chance that the enzymes have the same (or at least a very similar) mechanism.

By taking the unique combinations of CATH domains and incorporating sequence information (see Appendix and Fig. S1), we obtain a good approximation to the lower limit estimate. At this structural level, in which proteins were clustered according to their CATH domain combinations, we identified 1196 EC nodes (a fully defined EC number) that also had an associated structure deposited in the PDB (approximately one-quarter of all the currently defined EC nodes). We identified a total of 2244 structural clusters, which suggests that there are, on average, 1.88 distinct evolutionary families per EC node. It is interesting to note that the number identified by looking only at CATH domains is remarkably similar to that determined by using the sequence space for which there is also an associated PDB entry. Strikingly, the proportion of reactions performed by only a single family increases at this level to 66%, although the most diverse reaction is still the nonspecific Ser/Thr protein kinase (EC 2.7.11.1) reaction, with 58 evolutionary families at the structural level. It is unclear at this time exactly why the nonspecific Ser/Thr protein kinases are so diverse in terms of their protein families.

Although there are many EC nodes yet to be fully assigned in terms of both sequence and structure, we do not expect that the average number of families per EC node will change significantly, because, between 2009 and 2011, the average number of families per node changed by a value of 0.01, and almost 50 000 enzyme sequences were added to Swiss-Prot.

## Domain and subunit complexity in enzymes of known structure

Although biological activity requires the complete biological unit, not all of the protein structure may be catalytic, and so we can identify certain domains as catalytic and others as binding or noncatalytic. A catalytic domain can be defined as any CATH domain that furnishes at least one catalytic residue. An analysis of proteins annotated as enzymes in version 3.3 of CATH identified 1077 domains unique at the H level, of which only 411 distinct domains were catalytic in both MACiE and the CSA (which covers a total of 1056 unique enzymes).

**Fig. 3:**
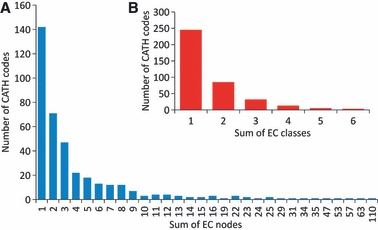
Bar charts showing the number of CATH codes (to the H level) associated with (A) the number of EC nodes and (B) the number of EC classes. An EC node is the EC number fully assigned to a serial number; for example, in A.B.C.D, the EC class is the first digit in the EC number, and represents (broadly speaking) the type of chemistry occurring. The catalytic domains have been identified as those that are catalytic in either MACiE or the CSA.

These domains can be further linked with the annotations of EC number through their assignment to specific PDB codes (which are assigned EC numbers). Although over one-quarter of the catalytic CATH codes are only represented by a single EC number (and therefore have a 1:1 mapping between CATH domain and overall reaction), there are some domains that are incredibly promiscuous with respect to the number of overall reactions in which they are involved (Fig. 3). The most promiscuous domain is 3.40.50.720, the NAD(P)-binding Rossmann-like domain, which is seen in 110 different EC numbers over all six classes of enzyme. Another example of a promiscuous domain is the trypsin-like serine protease domain (2.40.10.10), which is found in proteins catalysing 57 different overall reactions.

**Fig. 4:**
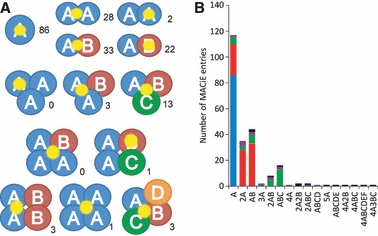
Domain complexity in enzymes. (A) Examples of the possible domain combinations of enzymes with a single domain, two domains, three domains and four domains; the yellow star represents the location of the active site, which can be in a single domain only, or at the interface between multiple domains, and the coloured circles represent the domains present, where the same colour and letter represents an identical domain. The numbers to the right of the image represent the number of entries in MACiE with that particular domain configuration. It should be noted that only a selection of the possible domain combinations have been shown here. (B) The full complexity of the catalytic domain configurations currently found in MACiE. The *x*-axis represents the different combinations found, and the *y*-axis represents the number of cases. For example, in the case of the single catalytic domain ‘A’, there are 86 enzymes that have only a single domain in the biological unit, 24 that have two domains in the biological unit, only one of which is catalytic, five that have three domains, and two that have four domains.

Whereas the biological unit is the assembly of domains and chains that is found in the cell and is required for full biological activity, we can further define a catalytic unit as the smallest assembly that is conceptually required for catalytic activity, i.e. the single unit containing the active site. Thus, a biological unit may have several catalytic units (and thus active sites). In the MACiE database, we only annotate a single catalytic unit, so we manually examined MACiE to identify the domain composition of the catalytic unit for each protein (to include the catalytic domains as well as the binding domains, identified as those that furnish at least three residues that are binding to a substrate, intermediate or cofactor). The different domain compositions of the enzymes in MACiE can be represented as shown in Fig. 4A, and although only a selection of the possible domain combinations is shown here, we have identified an enzyme that has nine catalytic domains, all of which are involved in catalysis (carbon monoxide dehydrogenase, EC 1.2.99.2, M0107), and the largest catalytic unit that we have identified to date contains 15 domains, two of which are involved in forming the active site (cytochrome *c* oxidase, EC 1.9.3.1, M0124).

More generally, only one-third of enzymes in MACiE are truly single-domain proteins in that they have a single domain in both their catalytic and biological assemblies. However, these single-domain enzymes span all EC classes of chemical reactions, although there does appear to be a slight preference for the hydrolases (EC 3) to be single-domain proteins (Table S2). Whilst MACiE does not yet include all enzymes, it is representative of enzyme space as defined by the EC classification [8]. However, it is still clear that a significant number of enzymes have multiple domains, as can be seen in Fig. 4B, which shows that, even though the catalytic unit of an enzyme might require only a single domain, the actual biological unit is composed of more than one domain.

A further complexity lies in the fact that an active site can be located in a single domain, at the interface between two domains, or even on multiple chains. Other arrangements include cases such as pyruvate dehydrogenase (EC 1.2.4.1, M0106), in which the catalytic site lies at the interface between two different chains, and although the catalytic domain is the same in each chain (CATH 3.40.50.970), one of the chains also has a second domain, which is not part of the active site. Thus, it can be seen that an enzyme is often composed of more than simply the active site or just the catalytic domains. Finally, it is worth noting that an enzyme can also have multiple active sites. For example, in glutamine-fructose-6-phosphate transaminase (EC 2.6.1.16, M0082), the first part of the reaction (the conversion of l-glutamine to l-glutamate and ammonia) is performed in one domain (CATH 3.60.20.10 on chain A) and the ammonia is then transferred through a channel in the protein to the second active site, where it is used in the second half-reaction (the conversion of d-fructose 6-phosphate to d-glucosamine 6-phosphate), which occurs at the interface between chains A and B, both of which have the same CATH code (3.40.50.10490).

## Conclusions

It is clear that, although we are steadily moving closer to a better understanding of enzymes and how they catalyse the chemical reactions required for the existence of life, there is still much complexity that we do not yet fully understand. This includes the diversity of chemistry performed by each amino acid, the diversity observed in associating a given reaction with a given protein, and the complexity of many enzymes in terms of their domain composition and quaternary structure.

Although many of the main catalytic amino acids (Arg, Asp, Cys, Glu, His, Lys, Ser, Thr, Trp and Tyr) perform most of the functions required for catalysis to occur, they do not do so uniformly between the different classes of enzyme [13]. It is also clear that cofactors add vital functionality to enzyme reactions, most notably by providing hydride shuttling for the organic cofactors, but also through being better at redox chemistry (metal ions) and by providing mechanisms for transferring groups (such as CO_2_ in biotin reactions) between different substrates [16].

Furthermore, enzyme databases such as MACiE provide an invaluable service by looking at the mechanism, as well as the overall chemistry, given that, simply because an enzyme is assigned the same EC number as another protein, there is no guarantee that the chemical mechanism will be the same.

Finally, the complexity of the protein structures, with respect to both the domain architectures and the chain combinations, poses significant computational difficulties, as well as posing interesting questions relating to the evolution of such complex entities. Enzymes are incredibly complex and beautiful, and we are only just beginning to scratch the surface of our understanding of them, for which tools such as MACiE are vital.

Although we have investigated the number of times that a specific function as defined by the EC node has evolved, we have not looked in detail at the evolution of function in general. Our own observations, along with work from the Babbitt laboratory [44], have demonstrated that evolution of function is incredibly complex and can follow many different routes, some of which include the following: (a) changes within the active site – such changes can lead to different overall chemistry via a similar mechanism [e.g. in the terpenoid synthase family (CATH 1.10.600.10), in which the same substrate is bound in many different conformations to produce a wide variety of different products], or the same overall chemistry via very different mechanisms [e.g. the fructose bisphosphate aldolase enzymes (MACiE M0052 and M0222, EC 4.1.2.13), in which a cyclic permutation changes the mechanism of the reaction, but not the overall reaction]; and (b) the domain combination of the enzymes may also change, and this may lead to changes in function (or complete loss of the original function) – a good example of the domain combination leading to different functions is provided by the Ntn-type amide hydrolase enzymes, which all have the CATH domain 3.60.20.10 in common; however, combination of this domain with a second, distinct domain changes the function of the enzyme, sometimes rather dramatically.

Although many such changes can be identified with the use of MACiE, they are beyond the scope of this review, which treats all EC nodes in isolation from one another.

The fact that, on average, an overall function appears to have evolved twice might be further evidence of divergence of function, in that similar catalytic apparatus can perform similar but distinct functions when presented with new situations, or indeed that domain combinations can be changed to change function. However, there are still many questions that are not fully answered, and this can only be achieved by combining sequence and structure with the chemistry, mechanism and phylogeny.

Over time, enzymes have evolved to be fit for purpose, and we see how nature has, on occasion, used multiple mechanisms to perform the same reaction or evolved a single protein family to perform many different reactions. This offers an insight into how we might proceed towards designing enzymes with novel functions, or how we might modulate the functions of known enzymes to perform specific tasks. However, we still need better tools with which to characterize and compare enzyme reactions, and we need better methods with which to elucidate and validate mechanisms. The MACiE database provides the basic data from which we can begin to obtain a better understanding of mechanisms and with which we will hopefully be able to predict and even design new mechanisms, given an enzyme sequence or structure.

## Abbreviations

CSA: Catalytic Site Atlas
EC: Enzyme Commission
PDB: Protein Data Bank

## Appendix

The appendix describes the methods used in the database analysis for determining the enzyme-to-reaction mapping for both the sequence and structure space.

### Calculating the number of evolutionary families by sequence

Each complete EC number (node) was determined from the IntEnz database [2]. We only included enzymes with a complete, four-digit EC numbers that have been manually curated (i.e. Swiss-Prot entries) [18] using the UniProtKB flat-files (the data were last downloaded in January 2011). Furthermore, we determined which of the sequences extracted also had deposited crystal structures (as determined by the Swiss-Prot annotation). Next, sequences containing fewer than 100 residues were discarded, as these are often fragments [45], and the remaining sequences were clustered into groups of homologues with the blastclust program, which is part of the NCBI blast package. blastclust uses blastp to determine the homology of two proteins, and uses single-linkage clustering, with a sequence similarity cut-off point of 35%. blastclust automatically and systematically clusters the protein sequences that it is given on the basis of pairwise matches found with the blast algorithm in the case of proteins. blastclust uses the default values for the blast parameters (matrix BLOSUM62; gap opening cost 11; gap extension cost 1; no low-complexity filtering), finds pairs of sequences that have statistically significant matches, and clusters them using single-linkage clustering. However, blastclust frequently generates a large number of singleton clusters with ‘orphan’ sequences, even with a low sequence identity cut-off point. Ideally, we would like as few ‘orphans’ as possible; thus, in order to place as many of these sequences with larger clusters, we utilized profile HMMs as implemented by hmmer 2 [46] to unify singleton sequences with other clusters where appropriate. Profile HMMs are statistical models of multiple sequence alignments that capture position-specific information about how conserved each column of the alignment is, and which residues are likely to occur at each column of the alignment. By searching an orphan sequence against an HMM profile generated on one of the clusters identified by blastclust, we can establish a likelihood of the sequence belonging to that cluster, and thus reduce the number of singleton clusters and the number of different possible nonhomologous families for a given EC number. When testing an orphan sequence against a profile HMM, we generated the average threshold score by utilizing a jackknife test, in which we systematically recomputed the threshold score of the HMM, leaving out one observation at a time from the sample set. From this new set of ‘observations’, we could calculate the average (mean) and standard deviation of the threshold scores for that model. The threshold score used for the singletons against an HMM model for a single cluster was then taken to be the mean plus two times the standard deviation. We also used an *E*-value cut-off of 1.0 (rather than the default 10), in order to ensure that we were not being too lax in our cluster reassignment, as our initial clusters were fairly loosely defined in the first place. Finally, we attempted to correct for those cases in which an enzyme is composed of nonhomologous chains, but for which both chains are required for catalytic activity to be present (e.g. pyruvate dehydrogenase, EC 1.2.4.1, M0106). However, this is only possible in those cases where we actually have PDB codes assigned to the enzymes. In these cases, where two (or more) different Swiss-Prot identifiers had the same list of PDB codes associated with them, we concatenated the clusters in which those Swiss-Prot identifiers occurred.

### Calculating the number of evolutionary families by structure

The dataset of Swiss-Prot sequences with fully assigned EC numbers and associated crystal structures was utilized in order to keep the results comparable with the analysis in the previous section. Initially, we simply collected all the PDB codes [19] associated with a sequence (in a chain-specific manner; that is, if Swiss-Prot identifier A lists only chain A as being associated with a given EC number, then only CATH codes relating to chain A are considered) and identified which of these had CATH codes assigned, using the latest version of the CATH database (version 3.3.0, released 7 July 2009 [47]). As in the upper limit calculation, each EC number was treated in turn and in isolation, and the unique combinations of CATH codes was determined. As previously noted [30], CATH classifies domains rather than entire proteins, so we decided to simply take unique combinations at face value; that is, if PDB1 contained the CATH code A.B.C.D and PDB2 contained the CATH codes A.B.C.D and W.X.Y.X, these two PDB files would be treated as two different groups, unless they belonged to the same Swiss-Prot identifier. This extra filter is added to ensure that extra domains attributable to protein substrates or the difference between the biological unit and the asymmetric unit do not significantly affect the results. Then, in order to ensure that differences in the biological unit reported for the PDB file were normalized, the list of Swiss-Prot identifiers were clustered by sequence similarity with blastclust (with the same parameters as were used for the upper limit calculation). However, it is worth noting that there may still be more than one grouping of enzymes that utilize CATH code A.B.C.D, but that are considered to be dissimilar in terms of the mechanism count, e.g. the class I (M0052) and class II (M0222) fructose-bisphosphate aldolases, which have an identical EC number (4.1.2.13) and CATH code (3.20.20.70), but which have distinct mechanisms. However, these two enzymes are indeed separated in the lower limit calculation by virtue of the fact that M0052 is a homodimer whereas M0222 is a homotetramer with nonidentical Swiss-Prot identifiers. Unfortunately, there is currently no method of automatically identifying the catalytic domain associated with a given EC number (except where the catalytic residues are known), or of identifying when an enzyme’s mechanism occurs across multiple domains in cases where there is more than one CATH code.

## Supporting information

The following supplementary material is available:

**Fig. S1.** The process of identifying first unique combinations of CATH domains for different proteins and then the second level of clustering, which takes into account the sequence identity in order to minimize the effect of domain ‘piggybacking’.

**Table S1.** The propensity of a residue to be catalytic in each of the six classes of enzyme.

**Table S2.** The coverage of single-domain enzymes with respect to the EC classification.

This supplementary material can be found in the online version of this article.

Please note: As a service to our authors and readers, this journal provides supporting information supplied by the authors. Such materials are peer-reviewed and may be re-organized for online delivery, but are not copy-edited or typeset. Technical support issues arising from supporting information (other than missing files) should be addressed to the authors.
