# Supplementary material for: Characterizing the complexity of enzymes on the basis of their mechanisms and structures with a bio-computational analysis
Source: FEBS J. 2011 Oct;278(20):3835–45. doi: 10.1111/j.1742-4658.2011.08190.x (PMC3258480; doi:10.1111/j.1742-4658.2011.08190.x)
Supplement: Supplementary file 1 [file febs0278-3835-SD1.pdf]

the FEBS Journal
www.febsjournal.org

Characterizing the complexity of enzymes on the basis of their mechanisms and structures with a bio-computational analysis

Gemma L. Holliday, Julia D. Fischer, John B. O. Mitchell and Janet M. Thornton

DOI: 10.1111/j.1742-4658.2011.08190.x

Supplementary Material

Propensity of an amino acid to be catalytic.

	Arg	Asn	Asp	Cys	Gln	Glu	His	Lys	Phe	Ser	Thr	Trp	Tyr
EC 1 Oxidoreductase	1.62	0.46	1.73	9.74	0.83	1.70	6.81	1.46	0.60	1.18	0.58	1.54	2.80
EC 2 Transferase	2.57	1.09	2.32	6.58	0.55	1.61	6.57	1.90	0.27	0.90	0.79	1.13	2.19
EC 3 Hydrolase	1.79	1.39	3.62	1.22	0.43	1.91	9.77	1.13	0.34	1.35	0.76	0.70	1.18
EC 4 Lyase	2.02	1.43	2.27	2.46	0.17	2.55	6.29	2.18	1.12	0.81	0.61	1.89	2.09
EC 5 Isomerase	2.04	0.24	1.98	5.54	0.27	1.63	5.23	2.08	1.56	1.12	0.19	2.87	4.28
EC 6 Ligase	1.94	-	2.42	2.37	2.10	0.29	8.23	5.93	-	0.98	0.94	1.95	-

Table S1: The propensity of a residue to be catalytic in each of the six classes of enzyme.

The data are taken from Version 2.5 of the MACiE database and are also available from the statistics and analysis section of the MACiE website (http://www.ebi.ac.uk/thornton-srv/databases/cgi-bin/MACiE/stats.pl)

EC Class analysis of the single domain proteins in MACiE V2.5

	Single Domain	Total Entry Count
EC 1 Oxidoreductase	21	78
EC 2 Transferase	14	51
EC 3 Hydrolase	36	66
EC 4 Lyase	19	43
EC 5 Isomerase	9	29
EC 6 Ligase	2	13

Table S2: The coverage of single domain enzymes with respect to the EC classification.

Calculating the number of evolutionary families by structure

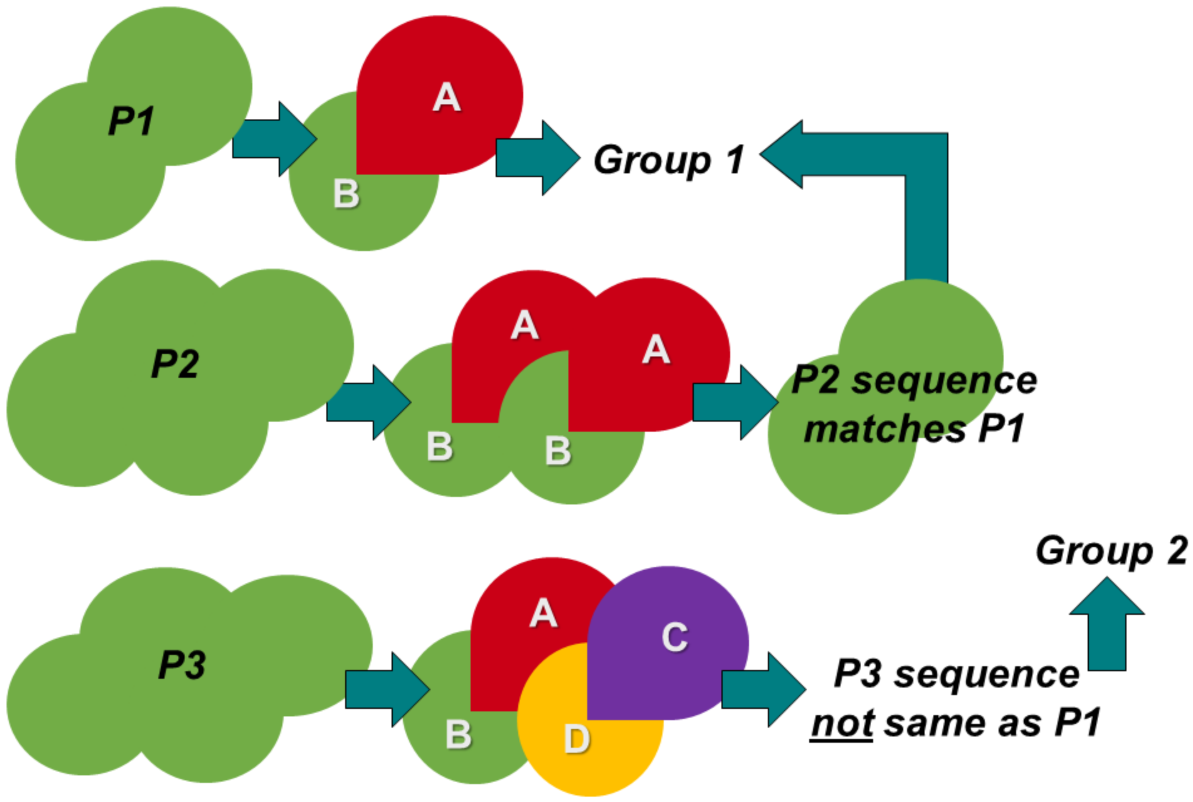

Figure S1: The process of identifying first unique combinations of CATH domains for different proteins and then the second level of clustering, which takes into account the sequence identity in order to minimise the effect of domain "piggybacking"
